# Single-blinded, stratified, dose ranging trial to assess pharmacokinetics and identify optimal dose of vitamin B12 in pregnancy in Tanzania

**DOI:** 10.12688/gatesopenres.15991.2

**Published:** 2025-09-17

**Authors:** Omar Lweno, Victoria S. Reynolds, Matthew D. Barberio, Kevin C. Klatt, Sabina Mugusi, Mathangi Gopalakrishnan, Zohra Lukmanji, Fadhlun M. Alwy Al-beity, Homa K. Ahmadzia, Amrita Arcot, Kelly Gallagher, Leigh A. Martin, Ali Rahnavard, Alison D. Gernand, Brooke Langevin, Honorati Masanja, Emily R. Smith

**Affiliations:** 1 Ifakara Health Institute, Dar es Salaam, Tanzania; 2 Department of Global Health, The Milken Institute School of Public Health, The George Washington University, Washington, District of Columbia, 20052, USA; 3 Department of Exercise and Nutrition Science, The Milken Institute School of Public Health, The George Washington University, Washington, District of Columbia, 20052, USA; 4 Department of Nutritional Sciences and Toxicology, University of California Berkeley, Berkeley, California, 94720, USA; 5 Department of Clinical Pharmacology, Muhimbili University of Health and Allied Sciences, Dar es Salaam, Tanzania; 6 Center for Translational Medicine, School of Pharmacy, University of Maryland Baltimore, Baltimore, Maryland, 21201, USA; 7 Department of Obstetrics and Gynecology, Muhimbili University of Health and Allied Sciences, Dar es Salaam, Tanzania; 8 Division of Maternal-Fetal Medicine, Department of Obstetrics & Gynecology, The George Washington University, Washington, District of Columbia, 20052, USA; 9 Division of Maternal-Fetal Medicine, Department of Obstetrics and Gynecology, Inova Health System, Falls Church, VA, 22042, USA; 10 Department of Nutritional Sciences, The Pennsylvania State University, University Park, Pennsylvania, 16802, USA; 11 Ross and Carol Nese College of Nursing, The Pennsylvania State University - University Park Campus, University Park, Pennsylvania, 16802, USA; 12 Computational Biology Institute, Departments of Biostatistics and Bioinformatics, The Milken Institute School of Public Health, The George Washington University, Washington, District of Columbia, 20052, USA

**Keywords:** Vitamin B12 Deficiency, Pregnancy, Controlled Feeding, Randomized Trial, Tanzania

## Abstract

**Background:**

Vitamin B12 is an essential cofactor for two enzymes that have critical functions in pregnancy, both for maternal health and fetal development. However, the optimal supplemental dosage and its correlation with vitamin B12 status during pregnancy remain inadequately understood due to limited data.

**Methods:**

This is a single-blinded, stratified, dose-ranging trial of vitamin B12 supplementation that will be conducted at the Ifakara Health Institute Bagamoyo Clinical Trial Unit in Bagamoyo, Tanzania. We will enroll 40 pregnant participants (gestational age 25–28 weeks) and 10 non-pregnant participants, stratified based on baseline vitamin B12 status (sufficient and insufficient). Pregnant participants are sequentially assigned to one of three doses: 2.6, 10, and 50 µg for four weeks. At the highest dose, pregnant participants are randomized to receive 50 µg once a day (Q24H) or 25 µg twice a day (Q12H). The two lower doses (2.6 and 10 µg) are given Q24H. Non-pregnant participants will receive 2.6 µg Q24H. The trial includes a four week in-patient phase for daily assessment and controlled feeding, with pregnant participants assessed once postpartum. Primary endpoints include serum B12 concentrations, holotranscobalamin concentrations, and their ratio after four weeks of daily supplementation.

**Discussion:**

This study aims to deepen our understanding of nutrient requirements in pregnancy by generating high-quality, high dimensional data. We will answer questions about how pre supplementation vitamin B12 status and dosage impact vitamin B12 saturable absorption and steady-state over the course of four weeks. Limitations include our inability to assess pharmacokinetic changes across gestation, the impact of vitamin B12 status or supplementation on pregnancy and fetal/newborn health, comparing vitamin B12 effects between pregnant and non-pregnant individuals above the recommended dietary allowance (2.6 µg), and comparing Q12H and Q24H dosing at 50 µg. This is the first controlled feeding study to be conducted in sub-Saharan Africa.

**Registration:**

ClinicalTrials.gov ( NCT05426395, 16/06/2022).

## Introduction

### Background and rationale

There is currently limited data regarding the pharmacokinetics of vitamin B12 and health outcomes related to maternal vitamin B12 status in pregnancy. It is important to understand the dose-response effects of vitamin B12 during pregnancy on nutrient and functional status markers and how maternal stores (baseline status) and gestational age modify this response. Although there are important knowledge gaps for many micronutrients in pregnancy, we have prioritized vitamin B12 for three reasons: (i) insufficient B12 intake is common in many lower middle-income countries (LMIC) ^1^; (ii) observational human data show that maternal vitamin B12 status is linked to adverse pregnancy outcomes ^2^; and (iii) emerging evidence suggests unique, pregnancy-related metabolic changes in functional vitamin B12 biomarkers (e.g. holotranscobalamin, homocysteine, methylmalonic acid ^3^), thus calling into question whether these biomarkers and their traditional cutoffs are appropriate for B12 deficiency screening during pregnancy.

B12 is well established as a cofactor for two enzymes including methionine synthase and L-methylmalonyl-CoA mutase. Functionally, B12 is crucial for red blood cell formation, neurological functioning, and DNA synthesis. Adequate maternal B12 status is critical in ensuring placental or mammary transfer of cobalamin to the developing fetus or infant ^1, 4^. B12 plays an important role during the perinatal period; it facilitates neural myelination, brain development, and growth of the offspring, alongside maintaining normal maternal functions ^2^. B12 deficiency causes megaloblastic anemia and related fatigue, weakness, and weight loss. It is often identified clinically by neurological problems including tingling or numbness in extremities, motor or visual disturbances, and cognitive problems (e.g., memory loss, disorientation, frank dementia) ^2^.

Subclinical B12 deficiency has recently been linked to several adverse maternal and neonatal health outcomes ^2^. Observational data show links between maternal B12 deficiency and adverse pregnancy and birth outcomes, including spontaneous abortion, pre-eclampsia, intrauterine growth restriction (IUGR), and low birthweight ^5^ For example, one study of 478 women in India showed an association between maternal B12 deficiency during pregnancy and increased risk for IUGR ^6^. Other studies have reported that elevated total homocysteine – typically observed when vitamin B12 status is compromised – is associated with low birth weight, independent of folate status ^7^. Low maternal B12 status has also been associated with adverse neonatal development including disruption of myelination and altered behavioral development, as well as disturbed inflammatory processes ^8^. There is evidence that infants born to mothers who maintain a vegan diet (and thus are likely B12 insufficient) are at increased risk of having low B12 stores at birth, and may develop overt signs of B12 deficiency within the first year of life ^9– 11^. However the existing body of evidence is based on low quality, observational data; evidence from high quality, prospective, and experimental studies are lacking ^2^.

While several studies in the 1960s-1980s suggest there are sex differences in average vitamin B12 serum levels ^12– 14^, there has been limited work to understand metabolism and special nutrient requirements for women in general, as well as pregnant and lactating women ^15^. Based on limited evidence, it is generally thought that: (i) dietary B12 absorption increases during pregnancy, though the degree to which it does is not known ^16^; (ii) recent maternal intakes, not maternal stores, are the primary source of vitamin B12 transferred across the placenta to the fetal compartment in a well-nourished context ^17^; (iii) serum total vitamin B12 concentrations decline throughout pregnancy, possibly due to plasma volume expansion, whereas the bioactive form of vitamin B12, holotranscobalamin (holoTC), remains unchanged in observational cohorts of uncomplicated pregnancy ^18, 19^. Little is known about changes in the efficiency of enterohepatic recycling and renal handling of vitamin B12 during pregnancy.

No experimental evidence of varying vitamin B12 intake in pregnancy was available for inclusion in the dietary reference intake (DRI) decision making process ^15^. The estimated average requirement and/or recommended dietary allowance (EAR/RDA) for vitamin B12 during pregnancy were established by increasing the DRIs for non-pregnant women by 0.2 µg/day to account for the estimated average rates of fetal deposition. There is currently no evidence regarding adverse events or serious adverse events related to high B12 intake.

Current nutrient reference values for B12 in pregnancy may be too low, even for women with otherwise adequate diets. One post hoc analysis of a controlled feeding study compared vitamin B12 metabolism in third-trimester pregnancy and lactation relative to non-pregnant women ^3^. Despite dietary (food and supplemental) vitamin B12 intakes in this study that were three times the RDA for pregnancy, pregnant women exhibited serum vitamin B12 levels in the low- to mid-range of the ‘normal’ reference range. Findings suggest that normal values may be different in pregnancy or that consumption of RDA levels remain insufficient even for this well-nourished population consuming diets meeting the DRIs. Higher B12 intakes in the study increased holotranscobalamin levels from baseline. The functional consequence of this remains unknown, though this observation conflicts with observational reports suggesting stable holoTC levels across pregnancy ^20^, suggesting this bioactive form of vitamin B12 is sensitive to higher intakes. Given that increased holoTC receptor availability has been observed in late pregnancy, it is critical to understand the relationship between vitamin B12 intake and holoTC, as this likely influences fetal stores ^20^.

### Objectives

We aim to: (i) enhance our understanding of vitamin B12 utilization and metabolic fate during pregnancy in people with sufficient (serum B12 concentration >220 pmol/L) and insufficient (serum B12 concentration ≤220 pmol/L) baseline B12 status; (ii) identify priority dose regimens of vitamin B12 that increase established vitamin B12 status indicators for investigation in later phase clinical trials to be conducted in populations where vitamin B12 insufficiency or deficiency is common; and (iii) identify novel biomarkers of vitamin B12 status appropriate for pregnancy.

### Reporting

The study protocol was developed and reported according to the SPIRIT (Standard Protocol Items: Recommendations for Interventional Trials) 2013 Guidelines ^21, 22^ and is available as extended data ^23^. The schedule of enrollment, interventions, and assessments is shown under Table 1.

**Table 1.** Schedule of enrollment, interventions, and assessments (SPIRIT Figure).

	STUDY PERIOD				
	Screening	Baseline	Post-allocation		Close-out
Timepoint	Day -8	Day 0	Day 1–28	Day 29	6-weeks postpartum
ENROLLMENT					
*Eligibility screen*	X				
*Informed consent*	X				
*Randomization / Allocation*		X			
INTERVENTIONS					
*2.6 ug B12*	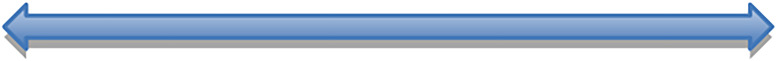				
*10 ug B12*	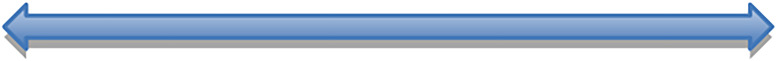				
*50 ug B12*	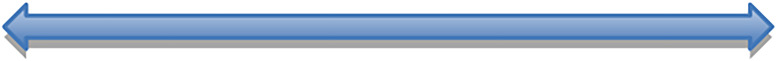				
ASSESSMENTS- MATERNAL					
*Hemoglobin*	X				
*Ultrasound*	X				
*Demographics*		X			
*Depression (PHQ-9)*		X			X
*Food frequency questionnaire*		X			
*Dietary recall*		X			
*Height*	X				
*Weight*	X	X	X	X	X
*Vital signs*	X	X	X	X	
*MUAC*	X	X	X	X	
*Blood sample*	X	X	X	X	
*Urine sample*	X	X	X	X	
*Breastmilk sample*					X
ASSESSMENTS- INFANTS					
*Stool sample*					X
*Weight*					X

### Trial design

This is a single-blinded, stratified, multiple ascending dose trial. Pregnant participants will be stratified based on their baseline serum B12 status. Women with a baseline serum B12 concentration >220 pmol/L will proceed into the “Sufficient B12 Status” strata and women with ≤220 pmol/L will proceed in the “Insufficient or Deficient B12 Status” strata ^10^. There are three ascending doses (2.6 µg, 10 µg, to 50 µg) that will be assessed in this trial. Each dose will have five participants assigned to it. Allocation to each dose group will be based on three factors: baseline B12 status, pregnancy status, and the timing of dosing (either every 12 hours or every 24 hours). Table 2 illustrates the dosing levels, timings, and stratification. We will begin with the lowest dose (2.6 µg) and complete the trial for participants in each group at this dose level before progressing to the next higher dose groups (10 µg to 50 µg).

**Table 2.** Trial Design. N=5 participants will be enrolled in each group.

			Dose 1 (2.6 µg)	Dose 2 (10 µg)	Dose 3 (50 µg)
**Sufficient B12 Status** serum B12 >220 pmol/L	Pregnant	Q12H	-	-	Group 3a
		Q24H	Group 1a	Group 2a	Group 3b
	Non-Pregnant		Group 1b	-	-
**Insufficient B12 Status** serum B12 ≤220 pmol/L	Pregnant	Q12H	-	-	Group 3c
		Q24H	Group 1c	Group 2b	Group 3d
	Non-Pregnant		Group 1d	-	-

Additionally, non-pregnant females will be enrolled and will be stratified based on their baseline serum B12 status. There will be no dose escalation or Q12 regimen arm in the non-pregnant group. Non-pregnant participants will only be assessed at the Dose I (2.6 µg), once per day (Q24H) level.

## Methods

**Tanzania Medicines & Medical Device Authority: TRC-WEB0023/CTR/-REG/0008, approved on May 3, 2023.**

## Participants, interventions and outcomes

### Study setting

In a previous study, the prevalence of vitamin B12 insufficiency among Tanzanian mothers in Dar es Salaam and Morogoro Regions was 25.6% three-months postpartum; insufficiency was found to be a key predictor of infant vitamin B12 deficiency ^24^.

Bagamoyo is a district located on the coast of Tanzania, approximately 60 km north of Dar es Salaam. The study will be conducted at the Ifakara Health Institute (IHI) Bagamoyo Clinical Trial Unit in Bagamoyo (BCTU) located on the Kingani Estate. The center has a track record of conducting good clinical practice (GCP) compliant phase 1, 2, and 3 studies including malaria sporozoite challenge studies, drug trials, and bioequivalence studies ^25– 27^. We modified the facility kitchen to serve as a metabolic kitchen that will be used for food preparation, refrigerated food storage, obtaining weighed food records, and dishwashing.

The trial, comprising both supplementation and follow up, will last for four weeks in total, with one additional follow-up visit for pregnant participants on or after 6 weeks postpartum. Participants will remain inpatient for the four weeks of the study to facilitate the controlled feeding study component and ensure direct observation of the intervention vitamin B12 supplement.

### Eligibility criteria

Research staff will assess eligibility criteria for pregnant and non-pregnant participants. The trial inclusion criteria for the pregnancy strata require that the participant is: (i) pregnant and female, (ii) has an estimated gestational age of 25 to 28 weeks at study initiation, (iii) is between the ages of 18 and 45 years of age, (iv) lives in the study area and does not plan to travel outside of the study area for the duration of the trial, and (v) consents to participate in the trial. The same eligibility criteria apply for those in the non-pregnant strata, less the pregnancy and gestational age requirements.

The 25-28 week gestation age window was chosen ^2, 18, 28^ to ensure that participants could complete the inpatient portion of the study before the end of pregnancy, and given preterm births may occur more commonly starting from 32 weeks gestational age, we set the maximum gestational age for enrollment at 28 weeks. It is also a time where physiological pregnancy changes are more pronounced. Longitudinal studies that have previously examined changes in Cobalamin status during pregnancy have demonstrated variations in B12 across the 2nd and 3rd trimesters. During this time, there is an increase in plasma volume causing hemodilution, decrease in the amount of carrier proteins, and maternal-fetal transfer of nutrients ^2, 18, 28^. Investigating the dynamics of B12 in the context of these physiological changes is of higher priority than the first trimester.

The trial exclusion criteria for pregnant participants include: (i) known multiple pregnancy (e.g. twins, triplets), (ii) severe anemia (hemoglobin <7 g/dL), (iii) pre-pregnancy or early pregnancy body mass index (BMI) ≥ 35 kg/m ^2^, (iv) self-reported pre-pregnancy history of type II diabetes mellitus, hypertension, or hypercholesterolemia, (v) currently diagnosed preeclampsia or eclampsia, (vi) currently diagnosed gestational diabetes, (vii) currently diagnosed renal, liver, autoimmune, or bleeding disorders, (viii) currently diagnosed congestive heart failure, (ix) a history of significant gastrointestinal surgeries, such as bariatric surgery, cholecystectomy, or other surgical procedures affecting the stomach, liver, bile ducts and/or small intestine that may disrupt enterohepatic recycling of vitamin B12, (x) a condition requiring the use of the following medications: H2 blockers, proton pump inhibitors, or prokinetic agents, (xi) regular use of an over-the-counter, high dose vitamin B12 supplementation, (xii) cigarette smoking or tobacco chewing, (xiii) heavy alcohol use (>3 drinks per day, or >7 drinks per week), (xiv) current malaria infection, (xv) HIV/AIDS infection, and (xvi) known allergy to corn or hydroxyethyl starch (HES). The trial exclusion criteria for non-pregnant participants are the same, except severe anemia will be defined as hemoglobin <8 g/dL and the pregnancy-related criteria (multiple gestation, gestational diabetes, and preeclampsia) do not apply.

## Ethics approval and consent to participate

The trial protocol was approved by George Washington University Institutional Review Board (Ref. No. NCR213648) on August 23, 2021, the Ifakara Health Institute Institutional Review Board (IHI/IRB/No: 47-2021) on November 9, 2021, the National Institute for Medical Research (NIMR) (Ref. No. NIMR/HQ/R.8a/Vol. IX/3963) on March 17, 2022, and the Tanzania Medicine and Medical Device Authority (TMDA) (Ref No. BD59/62/48/01) on July 5, 2022. Trained research staff will ask potential participants for written informed consent for trial enrollment as well as the use of their data and biological samples for future analyses.

### Who will take informed consent?

We will identify potential study participants among pregnant participants attending antenatal clinics at the Bagamoyo District Hospital and sentinel dispensaries within the study area. Interested women will be invited to a sensitization meeting to learn more about the study. Those expressing interest will undergo pre-screening for eligibility. Any women who meet the initial inclusion criteria based on the pre-screening assessment will be invited to provide informed consent before proceeding to the screening procedures. Trained research staff will ask potential participants for written informed consent for trial enrollment as well as the use of their data and biological samples for future analyses.

### Additional consent provisions for collection and use of participant data and biological specimens

In addition to participating in the study, study participants will be asked to provide consent for the collection and utilization of their biological specimens. This consent enables the staff to store blood samples for potential future research endeavors. If participants choose to withdraw their consent for the use of their blood samples in further research, they may simply inform any member of the study staff, prompting the removal or disposal of samples.

## Interventions

### Explanation for the choice of comparators

We selected 2.6 µg, 10 µg, and 50 µg doses based on several factors including the established nutrient reference value, doses used in previous pregnancy trials, and known pharmacokinetic properties of B12. The RDA set by the United States and Canada for vitamin B12 is 2.6 µg/day ^29^. In pregnant women, studies have used B12 doses ranging from 2.6 µg to 50 µg with no notable adverse events ^3, 30, 31^. One study in Bangladesh found that although pregnant women received a daily multivitamin with 2.6 µg B12 across pregnancy, there were still high rates of B12 deficiency (<150 pmol/L) in the third trimester compared to their early pregnancy, non-supplemented baseline ^29^. Established pharmacokinetic properties also influenced our dose selection. Vitamin B12 exhibits saturable dose-dependent absorption due to active processes involved in the absorption which limits bioavailability. On average, approximately 50% of a 1 µg dose and less than 5% of a 50 µg dose is absorbed ^1, 32^.

Given the saturable absorption and subsequent refractory period, we hypothesized that dividing the doses for more frequent, lower amounts could potentially achieve higher serum B12 levels as compared to one single dose at higher doses. A pre-clinical study in mice reported that more frequent dosing through diet better resolved low B12 status as compared to single dosing, mimicking supplementation ^31^. Thus, for this study the highest dose of B12 (50µg) will be evaluated as both Q24H dosing and Q12H dosing.

### Intervention description

We will provide the participants with vitamin B12 supplementation in the form of pharmaceutical-grade cyanocobalamin (DSM Nutritional Products Ltd, Lot No. 2109451822), first dissolved in sterile water to prepare a working stock solution, and then added to juice for consumption by participants in a blinded manner. The participants will be instructed to consume the juice in a 5- to 10-minute period, followed by consumption of 100 mL of purified water. Participants will be provided with daily juices to consume, regardless of their assigned regimen, to facilitate blinding regarding the specific vitamin B12 regimen. For individuals assigned to a Q24H arm, they will receive plain juice with added sterile water in lieu of the second B12 solution. Thus the second daily supplement will contain no vitamin B12. Vitamin B12 doses will be provided to participants within 30 minutes following the morning meal (and evening meal for Q12H dosing) and observed directly by pharmacy staff.

The intervention will occur in the context of a controlled feeding study where participants will be fed all food during their four-week inpatient stay from the on-site metabolic kitchen. All foods will be weighed on a gram scale with a 0.1g precision at all stages of food preparation, including raw ingredients, after cooking/preparation, and any returned/unconsumed food, to facilitate precise knowledge of the quantity prepared and consumed. A recipe database of culturally acceptable foods was developed and tested and integrated with the Tanzania Food Composition Tables to estimate nutritional contents ^31^. Meals will be planned on an individual basis for patients, meeting estimated energy and protein needs, and providing 2.6 micrograms of food-based Vitamin B12 per day from animal products (no fortified products were provided). Energy requirements will be assessed using standard regression equations and adjusted upwards to meet the energy demands of pregnancy and to provide an additional 100–200 kilocalories per day to limit potential for weight loss given due to individual requirements being higher than average and potential for unconsumed foods. Weights will be monitored weekly to determine weight gain adequacy, assessed relative to the mean 0.4kg per week expected weight gain in pregnancy ^33^. Insufficient weight gain will be assessed on an individual basis to determine likely etiology and adjust menus for the following week. Meal plans will be fixed for animal foods across participants to ensure similar Vitamin B-12 intakes and other animal food-based micronutrients, with energy and protein adjustments to meet individual requirements occurring through adjustment to planned quantities of low micronutrient starches. Standard operating procedures will be employed to ensure cleanliness of the metabolic kitchen and ensure safe food preparation and storage.

### Criteria for discontinuing or modifying allocated interventions

The study investigators can request a participant to stop their participation in the study at any time in the case that they are concerned for the participants’ safety, or if the participant is unable or unwilling to follow study procedures. If an investigator terminates an individual’s participation in the study, the investigator will explain to the participant the reasons for the termination. Participants may withdraw from the study at any time without compromising the quality of care provided at the antenatal clinic.

### Strategies to improve adherence to interventions

A strategy to improve adherence for our study intervention will involve administering the intervention under direct observation by the study pharmacist.

### Relevant concomitant care permitted or prohibited during the trial

Participants will be provided with iron and folic acid supplements as well as sulfadoxine-pyrimethamine for malaria prophylaxis as per standard of care during the antenatal period. 

### Provisions for post-trial care

No provisions for ancillary and post-trial care.

### Outcomes

The primary endpoints of the trial will be serum B12, holoTC, and the ratio of serum B12 to holoTC at day 29. A steady state pharmacokinetic analysis will be used to evaluate the accumulation ratio (steady state B12 level/baseline B12 level) and relative bioavailability between the three doses of B12. The steady state B12 levels will be evaluated descriptively between the three different doses (2.6 µg, 10 µg and 50 µg), different baseline B12 status (sufficient and insufficient), pregnancy status (pregnant and non-pregnant), and other subject specific prognostic factors.

Secondary outcomes will include: serum methylmalonic acid (MMA) at day 29; serum and urinary homocysteine at day 29; hematological response (hemoglobin, hematocrit, erythrocyte count, mean cell volume, reticulocyte number) at day 29. We will also assess the mean change from baseline to steady state B12 levels in pregnant participants for the three dose cohorts. The magnitude of difference (fold-change) in mean, baseline-adjusted steady state B12 levels between doses will be descriptively compared. Additionally, the proportion of participants who achieved or maintained sufficient B12 status will be assessed for each of the three dose cohorts. A dose with a higher fold difference (from 2.6 µg) and higher proportion of women having sufficient status will be identified as a priority B12 dose regimen. We will use metabolomics, proteomics, and genomics to identify novel biomarkers that can more robustly and sensitively reflect vitamin B12 status compared to conventional markers.

### Participant timeline

Within three weeks after screening and enrollment, the trial will begin (Day 0) for the participants. Weeks 1 to 4 (Days 1 to 28) is the inpatient phase and day 29 is the discharge day ( Table 3). Exit assessments take place on Day 29, and a follow-up visit will be scheduled postpartum. During the inpatient phase, participants will receive their assigned B12 dose every day and biospecimens will be collected as outlined in Table 3. On Saturdays, the participants can go home to visit their families for part of the day.

**Table 3.** Dosing & Sampling Frequency.

			Dosing	Sampling Frequency		
			B12 Supplement	Blood Sample	Fecal Sample	Urine Sample
Screening				X		
Enrollment	Day 0	Inpatient				
Week 1	Day 1	Inpatient	X	X	X	X
	Day 2	Inpatient	X	X	X	
	Day 3	Inpatient	X	X		
	Day 4	Inpatient	X	X		
	Day 5	Inpatient	X	X		
	Day 6	Home Visit	X	X		
	Day 7	Inpatient	X			
Week 2	Day 8	Inpatient	X	X		X
	Day 9	Inpatient	X			
	Day 10	Inpatient	X	X		
	Day 11	Inpatient	X			
	Day 12	Inpatient	X	X		
	Day 13	Home Visit	X			
	Day 14	Inpatient	X			
Week 3	Day 15	Inpatient	X	X		X
	Day 16	Inpatient	X			
	Day 17	Inpatient	X	X		
	Day 18	Inpatient	X			
	Day 19	Inpatient	X	X		
	Day 20	Home Visit	X			
	Day 21	Inpatient	X			
Week 4	Day 22	Inpatient	X	X	X	
	Day 23	Inpatient	X	X	X	
	Day 24	Inpatient	X	X		
	Day 25	Inpatient	X	X		
	Day 26	Inpatient	X			
	Day 27	Home Visit	X			
	Day 28	Inpatient	X			
Week 5	Day 29	Discharge	X	X	X	X

### Sample size

The main objectives of the Phase I dose escalation trial are to assess the pharmacokinetics (PK) of multiple ascending doses and to obtain information on the variability in serum B12 levels in pregnant participants after 4 weeks of supplementation. A total of 40 pregnant participants will be needed to precisely estimate the PK parameter (i.e., clearance) of B12 within 20% precision or the 95% confidence intervals within 60% and 140% of the geometric mean estimates of clearance at 90% power. The range of 60% to 140% for the 95% confidence intervals of clearance was based on FDA guidance for calculating sample sizes in pharmacokinetic (PK) studies ^34^. We estimated the between-subject variability of vitamin B12 in PK parameters to be between 50% and 70% ^3, 30, 31^ . We used the methodology for sample size calculation based on Wang *et al*. ^35^. We determined that 21–24 subjects would be required to achieve 90% power to precisely estimate the PK parameters. To account for potential attrition and ensure sufficient representation for each subgroup, the sample size was increased to 40 pregnant subjects. The 40 pregnant participants will be stratified based on their baseline B12 status and assigned to the three B12 dose groups such that there are 5 participants per dose group per strata ^36^. Additionally, 10 non-pregnant participants will be enrolled to serve as the reference group to understand potential differences in background B12 status in the Tanzanian population. The target sample size will therefore be 50 participants, including 40 pregnant and 10 non-pregnant participants.

### Recruitment

**Figure 1.**
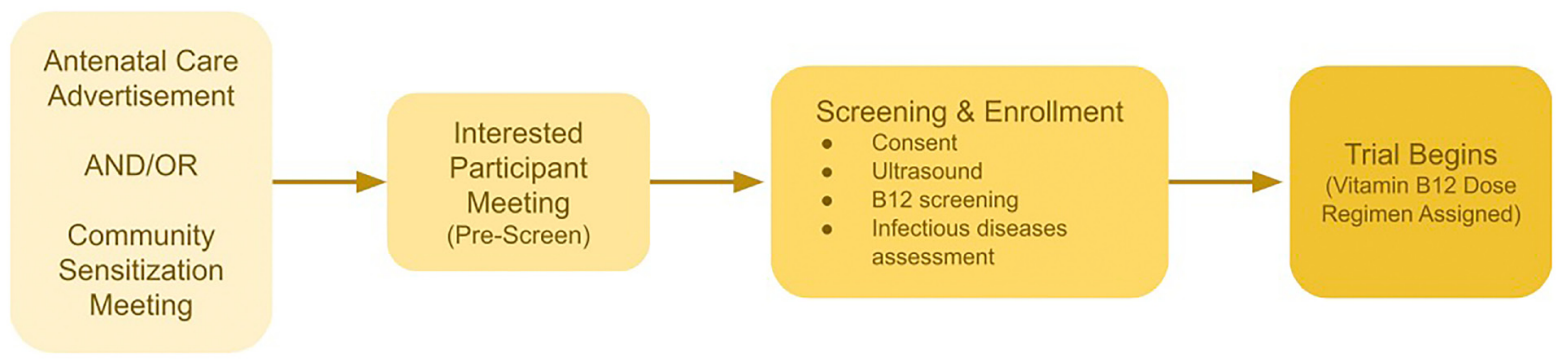
Recruitment, Screening, and Enrollment Process.

Recruitment and enrollment are a multi-stage process ( Figure 1). We will recruit potential study participants among pregnant participants attending antenatal clinics at the Bagamoyo District Hospital and sentinel dispensaries in the study area. Information related to the study will be provided in different stages, starting with advertisements in the antenatal clinic waiting areas and presentations by nurses in charge of the clinics. Research Nurses will invite interested women with estimated gestational age <28 weeks, together with their spouses or partners, to a community sensitization meeting involving community health workers, study investigators, and members of the community advisory board (CAB). During the first meeting, the research team will explain the problem of malnutrition among pregnant participants and young children, current prevention strategies and their limitations with a focus on vitamin B12. We will discuss the need to have well-controlled feeding studies to guide future interventions of micronutrient deficiencies in pregnant participants, as well as a description of the objectives and design of the proposed clinical trial. Particular attention will be paid to study procedures like screening of eligible participants, adherence to protocol-specified diet, duration of in-facility observation, and timing of collecting samples for laboratory analysis. The meeting will be conducted in Swahili at designated community halls in the study area. Each couple attending this meeting will be provided with a copy of the participant information sheet (PIS).

Any interested participants will be invited to a second meeting; they will be listed in the pre-screening log and invited to the Kingani Clinical Trial Facility. The second session will provide a detailed explanation of the study, rationale, objectives, and methods. The potential study participants will be provided with an opportunity to ask questions and receive responses from the study investigators. Women who are still assessment will be invited to give informed consent before interested in participating will be pre-screened for eligibility. continuing the screening procedures. Consented women will Any women who are likely eligible based on the pre-screening then be invited to complete an ultrasound and give a blood sample for serum B12 assessment. They will be given appointments to start the trial when they reach the protocol defined gestational age at recruitment. We expect the whole process from sensitization meeting to confirming enrollment eligibility and consent will take 1–2 weeks. We note that there is potential selection bias in this recruitment method, and the study may be more likely to attract women who are not formally employed, who do not have other children at home, or who are single. However, such bias is unlikely to alter the biological mechanisms that we focus on in this study, specifically vitamin B12 pharmacokinetics, absorption, and metabolic parameters.

## Assignment of interventions: allocation

### Sequence generation

The B12 pharmacokinetic study includes two strata: sufficient B12 status and insufficient B12 status. Within each strata, the study participants will be allocated to three B12 doses: 2.6 µg, 10 µg, and 50 µg in sequential, ascending order. Within each strata, dose 1 (2.6 µg) will be allocated to pregnant and non-pregnant participants. For the 50 µg dose only, randomization to Q12 or Q24 regimens will occur at enrollment. The study pharmacist will use a computer-generated list to randomly assign participants to the once or twice daily regimen. A list with numbers from 7777 through 8888 will be prepared according to a randomization sequence by the study statistician. The screening ID will be linked to the randomization ID by the pharmacist on site. The randomization code will have an “x” digit, the first digit indicating the study group and the following digits indicating the participant number within that group. In total, there will be 10 subgroups each with 5 participants.

### Concealment mechanism

The dose assignment is maintained by the study statistician and pharmacist. The list is maintained in the locked pharmacy where only authorized personnel may enter.

### Implementation

For the study implementation, the allocation sequence will be generated by the study statistician. Participants will be enrolled in the study by the study clinician, while assignment to the intervention will be conducted by the study pharmacist.

## Assignment of interventions: blinding

### Who will be blinded

The participants will be blinded to their dose level and the fact that the doses are provided in ascending order. Study staff responsible for data collection will also be blinded to the B12 dose regimen. The study pharmacist responsible for preparing the supplement and directly observing supplement administration, as well as study investigators, will not be blinded.

### Procedure for unblinding if needed

Not applicable.

## Data collection and management

### Plans for assessment and collection of outcomes

On the screening day, we will screen for infectious diseases using rapid diagnostic kits as currently used by the Government of Tanzania. These screening tests will include malaria, syphilis, hepatitis B, hepatitis C, and HIV. Additionally, we will assess participants for parasitic infections including soil-transmitted helminthiasis (STH) and schistosomiasis. On the screening day, enrollment day, and every day during the inpatient phase (days 1 to 29), we will conduct clinical assessments including measurements of weight and blood pressure. A micronutrients assessment panel will be done on the day 1, day 22, and day 29 and will include the following biomarkers: iron (serum ferritin, soluble transferrin receptor), vitamin A (retinol binding protein 4), and iodine (thyroglobulin). We will also assess biomarkers of inflammation including C-reactive Protein (CRP) and α-1-acid glycoprotein (AGP). We will assess proteomic, metabolomic, and metagenomics on days 1 and 29, as well as days 8, 21, and 22 for metabolomics.

Throughout the trial, we will assess vitamin B12 status using multiple biomarkers including serum B12 and holoTC, which will be measured during the inpatient phase (screening day, days 1–6, day 8, day 10, day 12, days 14–15, day 17, day 19, days 21–29); and methylmalonic acid (MMA), total homocysteine (tHcy), Haptocorrin–holo, Haptocorrin–total that will be measured at: baseline (screening day), days 1–2, and subsequent Mondays (days 8, 15, 22, and 29).

### Plans to promote participant retention and complete follow-up

Throughout the inpatient phase of the study, we will promote retention by ensuring participants are comfortable, well-fed, and adequately entertained. We will organize television and movie time, physical activities, and educational activities.

To promote retention in the six weeks postpartum follow-up visit for pregnant participants, research staff will request the participants to inform the study team when they deliver the baby, or reach the endpoint of the pregnancy otherwise. Upon receiving such notification, the research staff will then schedule one final visit 42 days within the endpoint of the participant’s pregnancy. If the research staff do not hear from the participant two weeks after their estimated due date, the staff will follow up with them via phone call to inquire about the status of their pregnancy.

### Data management

The trial data management team is responsible for overseeing the receiving, entering, cleaning, querying, analyzing and storing all data that accrues from the study. Trained study staff will input participant data into electronic Case Report Forms (CRFs) for specific components such as the screening form, enrollment data, baseline and demographic information, food frequency questionnaire, 24-hour dietary recall, ultrasound details, plasma volume measurement, clinical records, daily food records, daily B12 dosing records, daily maternal vital signs and GI complaints, postnatal follow-up documentation, adverse event log, protocol deviation log, withdrawal termination forms, and antenatal pregnancy health records.

Laboratory sample requests for prescreening, screening, and routine daily testing, as well as the recording of all lab results data, will be initially documented using paper-based forms. Subsequently, this data will be entered into electronic databases for efficient management, sharing, and analysis purposes. All source documents and laboratory reports will be reviewed by the clinical team and signed by the study lead clinician and data manager who will ensure that they are accurate and complete.

The electronic data capture system will be developed in REDCap, a secure and web-based application used for streamlined data collection and management in research studies. Data entry into the 21 CFR Part 11-compliant Electronic Data Capture system will be carried out by study staff. Clinical data will be entered directly from the source documents. Direct access to the study database will be granted to authorized representatives from implementing teams and regulatory authorities to permit trial-related monitoring, audits, and evaluation of the study safety, progress, and data validity.

All source documents will be filed in participant files including: volunteer consent form, clinician’s medical notes, laboratory test results, and relevant correspondences. In the majority of cases, electronic CRF entries will be considered source data because it is the original recording of the information (i.e., there is no other written or electronic record of data). If participants fall ill and receive medical treatment, notes and investigational results will be considered as source documents and stored in participant files. All source documents will be stored safely in a locked filing cabinet where only authorized study investigators will have access. Only anonymized study data will be shared with third parties including study sponsors.

Study documents will be retained for a minimum of 20 years after the end of the clinical trial. No records will be destroyed without the written consent of the PI, and it is the responsibility of the PI to inform the investigator when these documents no longer need to be retained.

### Confidentiality

The investigators will maintain appropriate medical and research records for this trial in compliance with ICH E6 GCP and regulatory and institutional requirements for the protection of confidentiality of participants. The investigators will permit authorized representatives of regulatory agencies and the monitors to examine clinical records for the purposes of quality assurance reviews, audits and evaluation of the study safety and progress.

The names of the participants and any other identifying detail will not be included in any trial data electronic file. On all trial-specific documents, other than the signed consent, the participant will be referred to by the trial Study ID number, and not by their identifying information.

### Plans for collection, laboratory evaluation and storage of biological specimens for genetic or molecular analysis in this trial/future use

Whole blood samples will be collected throughout the duration of the study to assess B12 status, B12 biomarkers, micronutrient status, and inflammatory biomarkers. Table 3 outlines the sampling schedule. Specifically, for blood samples, one blood draw of 6 total mL (~0.4 tablespoons) will take place on the following days: screening day, days 4–6, 8, 10, 12, 15, 17, 19, 25, 29. Each 6 mL will be drawn into serum separator (4 mL SST) and plasma (2 mL K _2_-EDTA) blood collection tubes (Becton, Dickinson, and Co., Franklin Lakes, NJ). On days 2–3 and 23–24, two blood draws of 6 mL each will be taken. On day 1 and day 22, serum samples will be collected before supplement intake and at 14, 24, and 48 hours post-dose. These samples will be obtained by drawing 3 mL of whole blood into serum separator tubes. Additionally, on day 1 and day 22, plasma samples will be collected at pre-dose, 6, 14, and 24-hour intervals by drawing 3 mL of whole blood into K2-EDTA collection tubes. An additional 3 mL plasma sample will be collected on days 1 and 22, respectively, for plasma volume measurement.

Plasma volume measurements will be conducted approximately 30 minutes before consumption of the morning meal and supplement. Participants should be fasted for >8 hours (no food) but continuing to drink water. Women will rest in a supine position on their left side for 10 minutes before insertion of an IV in an antecubital vein. They will rest for 5 minutes after IV insertion. After the rest period, a pre-injection blood draw will be conducted to collect whole blood in one 3 mL K _2_-EDTA blood collection tube. Thereafter, 170 mL of hydroxyethyl starch (HES) solution (HESPAN: 6% Hetastarch in 0.9% Sodium Chloride, B.Braund Medical Inc., L6511) will be injected through the IV catheter over 4 minutes followed by a flush with ~10 mL saline. A single post-HES injection blood sample of 3 mL in a K _2_-EDTA blood collection tube will be taken 10 minutes after completion of the HES injection.

Twenty-four hour urine samples will also be collected 1 day per week on days 1, 8, 15, and 29. Participants will be given a sample collection jug and asked to use it for all urine voids. Samples will be mixed thoroughly before collecting 50 mL for storage. Fecal samples will be collected on Day 1–2, 22–23, and 29. All stool samples on these days will be collected and stored individually in 5 mL collection tubes. Upon collection, samples will be thoroughly mixed before aliquoting into storage vials. Samples will be immediately frozen at -80 degrees celsius.

For pregnant participants, breast milk samples will be collected once at the 6 weeks postpartum visit. Approximately 10 cc of breast milk will be obtained through full breast expression. The samples will be collected by a pump, stored individually in 10 mL collection tubes and immediately frozen at -80 degrees celsius. The outcomes to be evaluated include the concentration of vitamin B12 and its metabolites, micronutrients and trace elements in human milk, the human microbiome, and multiomics analysis. Infant fecal samples will also be collected once at 6 weeks postpartum visit. All stool samples on these days will be collected and stored individually in 5 mL collection tubes. Samples will be thoroughly mixed before aliquoting into storage vials and immediately frozen at -80 degrees celsius.

Blood, urine, and fecal samples will be processed and stored long term in the on-site laboratory. At specified times, a small portion of plasma and serum samples will be used to conduct infectious disease (Screening Day) and clinical testing (Screening Day, Day 1, 8, 15, 22, and 29). On Day 1 and 22, whole blood (from a K _2_-EDTA blood collection tube) will be used for a complete blood count. Whole blood will then be processed for plasma according to manufacturer instructions: blood collection tubes will be centrifuged at room temperature and 1000 to 1300 RCF for 10–15 minutes. Plasma will be aliquoted into ~500 uL aliquots for plasma volume measurement and storage at -80 degree celsius for later laboratory analysis as needed.

Plasma volume assessment will occur on-site the same day of HES injection, or conducted from frozen plasma if same-day measurement is not possible. Glucose will be measured in the pre- and post-HES injection plasma samples using a hand-held glucometer. Laboratory procedures for processing the post-injection plasma have been described ^37^. Samples are centrifuged and glucose is measured in the supernatant. Results will be entered into an equation to calculate plasma volume.

## Statistical methods

### Statistical methods for primary and secondary outcomes

The primary focus of the analysis is to determine the most optimal dosage of vitamin B12 for pregnant participants with varying baseline levels of B12. Through descriptive analysis, we aim to explore dose-response relationships to inform future research.

For the primary analysis, steady state pharmacokinetic analysis will be used. The main objective of the analysis is to assess the steady levels of B12 for the different dose cohorts (2.6μg, 10μg and 50 μg) in pregnant participants. The data for the pharmacokinetic analysis will include the sparse B12 concentrations (dose: 2.6μg, 10μg and 50μg (both once daily and twice daily)) measured at multiple occasions over the 4-week time period. The pharmacokinetic analysis will involve two steps: (i) descriptive and graphical analysis (ii) steady state pharmacokinetic analysis to evaluate the accumulation ratio (steady state B12 level/baseline B12 level) and relative bioavailability between the three doses of B12. The steady state B12 levels will be evaluated descriptively between the three different doses (2.6μg, 10μg and 50μg), different baseline B12 status (sufficient and insufficient), pregnancy status (pregnant and non-pregnant) and other subject specific prognostic factors. To determine whether steady state of B12 has been achieved or not, individual and mean longitudinal graphs of pre-dose serum B12 levels over time across the three dose cohorts will be used. Presence of a flat profile over a period of time would indicate achievement of steady state. Additionally, the pre-dose B12 levels on three days during week 4 will be regressed over time and will be tested for the slope to be not different from zero. A mixed effects linear regression will be performed to assess the significance of slope. If the 95% confidence interval of slope would contain zero, then achievement of steady state will be made. 

As a secondary analysis, the following analyses will be conducted. Once the steady state is ascertained, from the mean change from baseline steady state B12 levels in pregnant participants for the three dose cohorts, magnitude of difference (fold-change) in mean change from baseline steady state B12 levels between doses will be descriptively compared. The mean change from baseline steady state B12 will be descriptively compared between the following subgroups namely by pregnancy status and other clinically relevant prognostic factors. Steady state levels will be utilized as surrogates for clearance to aid in selecting doses for future trials. The relative bioavailability will be calculated as relF = (dose/ mean steady state B12 for dose)/(dose = 2.6µg/mean steady state B12 for 2.6µg). Additionally, proportion of participants who achieved or maintained sufficient B12 status will be assessed for each of the three dose cohorts. A dose with a higher fold difference (from 2.6µg) and higher proportion of women on sufficient status will be identified as a priority B12 dose.

To facilitate discovery of biomarkers associated with vitamin B12 intake, bioavailability, and metabolic outcomes in pregnancy, we plan to apply high throughput -omics approaches and associated bioinformatic pipelines to plasma samples derived from this study. Fecal samples procured throughout the trial (Day 1, 22, 29) will be subjected to shotgun metagenomic and metatranscriptomic sequencing, facilitating characterization of the functional profiles of present microbial communities in an unconstrained manner. High throughput metabolomics and proteomics will be facilitated by the Bill & Melinda Gates Foundation Global Health Platform Partners, Sapient Bioanalytics, LLC and Cedars-Sinai Precision Biomarker Laboratory, respectively. Microbial genome and transcriptome sequencing will be undertaken at the Broad Institute. Bioinformatic and statistical analysis of this data will be undertaken in the laboratory of the Dr. Ali Rahnavard, utilizing both existing and novel pipelines, to identify: (i) novel correlates of vitamin B12 intake and status; (ii) predictors of vitamin B12 bioavailability; (iii) predictors of the change in vitamin B12 status indicators and plasma volume expansion across pregnancy. Collectively, these multi-omic techniques will help to elucidate novel modifiers of nutrient metabolism during pregnancy and inform future investigations.

### Interim analyses

The serum B12 results will be reviewed with the DSMB at the completion of each dose cohort. There are no stopping rules for the study.

### Methods for additional analyses (e.g. subgroup analyses)

Not applicable.

### Methods in analysis to handle protocol non-adherence and any statistical methods to handle missing data

While imputation techniques might not be suitable for our specific context, our analysis strategy is tailored to optimize available data. Participants with baseline B12 measurements and at least one subsequent B12 assessment will form the basis of our analysis cohort. In instances where B12 data are absent at particular time points for a participant, we will adopt a conservative approach by excluding those specific time points from the individual's analysis. This ensures that our analytical methods remain robust and reflective of the available data, thereby maintaining the integrity and reliability of our findings within the study framework.

### Plans to give access to the full protocol, participant level-data and statistical code

The datasets generated from the trial may be made available from the corresponding author on reasonable request and approval from applicable Institutional Review Boards.

## Oversight and monitoring

### Composition of the coordinating centre and trial steering committee

The international trial management team will meet weekly to review study progress, data quality, and any issues that arose during the past one week. Additionally, the implementing clinical team will meet every Monday in person and every Thursday virtually, that involves collaborators in the US. The controlled feeding kitchen team will meet weekly to review menus and discuss necessary modifications needed to meet participant requirements/requests.

### Composition of the data monitoring committee, its role and reporting structure

The DSMB members will review primary outcome data after each dose cohort completes the inpatient phase of the trial. We will inform the DSMB of any severe adverse events within 48 hours. There are no specific stopping rules for this trial. Additionally the DSMB will review the protocol for scientific validity and any major concern prior to implementation, review study data to evaluate safety, quality of study conduct, and scientific integrity of the trial, and assess the performance of overall study operations. Additionally, a clinical trial monitor appointed by the sponsor, IRB committee and TMDA will intermittently monitor the conduct of the trial.

### Adverse event reporting and harms

The potential side effects of vitamin B12 supplementation will be closely monitored throughout the study. Adverse events (AEs) and side effects, including gastrointestinal symptoms and allergic reactions, will be assessed on a daily and weekly basis. These will be reviewed regularly to ensure participant safety. A serious adverse event (SAE) will be defined as any undesirable experience associated that is life-threatening or requires inpatient hospitalization. In this study, SAEs include death, life-threatening events (at substantial risk of dying at the time of the adverse event), inpatient hospitalization (initial or prolonged), and stillbirth. Any SAE will be reported to the Data and Safety Monitoring Board within 48 hours of reporting, as well as subsequently reported to the local safety monitor, and all ethics committees including those at The George Washington University, the Ifakara Health Institute (IHI), and the National Institute for Medical Research (NIMR). Given that the dosages of 2.6 mcg, 10 mcg, and 50 mcg of vitamin B12 used in this trial have been previously tested in pregnant populations with no significant side effects reported ^30^, we will monitor participants closely for any adverse events and adjust the protocol as necessary to ensure participant safety.

### Frequency and plans for auditing trial conduct

The study monitor will complete four clinical monitoring visits and a final close-out visit. Additionally, TMDA and IHI IRB are likely to conduct at least one oversight visit during the course of the trial.

### Plans for communicating important protocol amendments to relevant parties (e.g. trial participants, ethical committees)

Any protocol modifications will be addressed and communicated to relevant parties (investigators, IRBs, trial participants, trial registries, TMDA) through written communication via email.

### Dissemination plans

This study has potential benefits at the population level, and the results may also benefit study participants and local communities. Therefore, data will be aggregated and summarized for publications and presentations. During the study (after unblinding process) and upon completion of the trial, we will publish key research findings in peer-reviewed journals and scientific conference abstracts with suitable audiences. We will present selected results at conferences, research seminars, and meetings with a focus on nutrition in the global health context.

We will also relate the findings back to the study participants and local communities using several approaches and guided by the community advisory board. For the study participants and local communities, we also plan to engage the study participants in a post-trial, voluntary focus group discussion. Through this focus group, we aim to understand their experiences as participants, gauge their understanding of the benefits and goals of the trial, and to glean feedback about how to improve future implementation of studies. After the focus group discussion, we will invite the local community members to join a meeting, during which we will share highlights of the study through science communication techniques. We will foster an interactive session to ensure that the key points are understood through a question and answer session at the end. We will also encourage community leaders to share feedback regarding how we may improve future studies to further benefit local communities. For local research partners, we will hold one research seminar at IHI to disseminate the results to the broader research community in Tanzania. 

## Discussion

This study is intended to inform our basic understanding of nutrient requirements in pregnancy. We will answer important questions about how vitamin B12 dose and status prior to supplementation affect saturable absorption and steady-state B12 status over the course of four weeks. We will provide preliminary data to answer the question about whether twice daily dosing, as compared to once daily dosing, can achieve a higher steady-state B12 status. We will also generate the first high quality pharmacokinetic data comparing pregnant and non-pregnant participants (with both sufficient and insufficient baseline vitamin B12 status) in the same population. To our knowledge, this will be the first controlled feeding study conducted in sub-Saharan Africa.

There are several limitations to this study. First, the study design requires stratifying participants by sufficient and insufficient vitamin B12 status at baseline. Thresholds for defining sufficient and insufficient status in pregnancy are not well established. Further work is needed to establish the clinical value of these thresholds in pregnancy. Further, we will not answer questions about how pharmacokinetic properties change over the course of pregnancy. We will not answer questions about how B12 status or vitamin B12 supplements impact pregnancy health or fetal/newborn health. We also cannot answer questions about the effect of vitamin B12 supplements and how pharmacokinetic properties compare between pregnant and non-pregnant people at doses above the RDA (2.6 µg) or how twice daily dosing compares to once daily dosing at doses below 50 µg.

## Trial status

The protocol version of MM4MN B12 Dose Escalation Trial: Phase 1 is on Protocol version 3.7 dated January 16, 2023. Recruitment began May 29, 2023. The phase 1 trial enrollment was completed on December 18, 2023 and participant follow up and data collection is still ongoing.

## Ethics approval and consent to participate

The trial protocol was approved by George Washington University Institutional Review Board (Ref. No. NCR213648) on August 23, 2021, the Ifakara Health Institute Institutional Review Board (IHI/IRB/No: 47-2021) on November 9, 2021, the National Institute for Medical Research (NIMR) (Ref. No. NIMR/HQ/R.8a/Vol. IX/3963) on March 17, 2022, and the Tanzania Medicine and Medical Device Authority (TMDA) (Ref No. BD59/62/48/01) on July 5, 2022. Trained research staff will ask potential participants for written informed consent for trial enrollment as well as the use of their data and biological samples for future analyses.

## Consent for publication

Not applicable.

## Abbreviations

**Table:** 

AIDS AGP	Acquired Immunodeficiency Syndrome α-1-acid glycoprotein
BCTU BMI	Bagamoyo Clinical Trial Unit Body Mass Index
CAB CRF CRP	Community Advisory Board Case Report Form C-reactive Protein
dL DNA DRI DSMB	deciliter Deoxyribonucleic Acid Dietary Reference Intake Data Safety and Monitoring Board
EAR EDTA	Estimated Average Requirement EthylenediamineTetraacetic Acid
GCP GW g	Good Clinical Practice George Washington University Grams
HES HIV H2	Hydroxyethyl Starch Human Immunodeficiency Virus Hydrogen
ICH ID IHI IRB IUGR IV	International Council for Harmonization Identity Ifakara Health Institute Institutional Review Board Intrauterine Growth Restriction Intravenous
kg	Kilogram
L LMIC LMP	Liter Lower Middle-Income Countries Last Menstrual Period
m mL MMA	meter Milliliter Serum Methylmalonic Acid
NIMR	National Institute of Medical Research
PI PIS PK pmol	Principal Investigator Participant Information Sheet Pharmacokinetic Picomoles
Q12H Q24H	Twice Daily; Every 12 Hours Once Daily; Every 24 Hours
RDA	Recommended Dietary Allowance
SAE SST STH	Serious Adverse Event Serum Separator Tube Soil-transmitted helminthiasis
tHcy	Total Homocysteine
µg μL	Microgram Microliter

## Data Availability

No data are associated with this article. Repository: SPIRIT checklist for ‘Single-blinded, stratified, dose ranging trial to assess pharmacokinetics and identify optimal dose of vitamin B12 in pregnancy in Tanzania. https://doi.org/10.17605/OSF.IO/GRVN4 ^23^ Data are available under the terms of the Creative Commons Zero "No rights reserved" data waiver (CC0 1.0 Public domain dedication)
