# Supplementary material for: Single-blinded, stratified, dose ranging trial to assess pharmacokinetics and identify optimal dose of vitamin B12 in pregnancy in Tanzania
Source: Gates Open Res. 2025 Sep 17;8:95. Originally published 2024 Sep 4. [Version 2] doi: 10.12688/gatesopenres.15991.2 (PMC12759048; doi:10.12688/gatesopenres.15991.2)
Supplement: Peer review history

# Reviewer report: Reviewer response for version 2

**DOI:** 10.21956/gatesopenres.17759.r39958

**Published:** 2026-01-02

**Authors:** Kristina Pentieva

**Affiliations:** 1 Ulster University, Ulster, UK

The protocol outlines a single-blinded, dose-ranging trial of vitamin B12 supplementation in pregnant (gestational age 25–28 weeks) and non-pregnant Tanzanian women and it is aiming to generate data for the metabolic fate of B12 in pregnancy, to identify the supplemental dose required for optimization of B12 biomarkers as well as to identify novel biomarkers of vitamin B12 status relevant for pregnancy. The trial will be conducted under strictly controlled conditions in a Clinical Trial Unit where the participants will be kept inpatient for the duration of the supplementation. Based on baseline vitamin B12 status participants will be stratified (sufficient and insufficient) and depending on their reproductive state will be assigned for four weeks to the following B12 supplementation regimes: 1). Pregnant: sequentially to one of three doses: 2.6, 10, and 50 μg/day; 2). Non-pregnant: 2.6 μg/day. The study is carefully designed according to the rules for such trials and has the potential to generate valuable new data in relation to pharmacokinetics of vitamin B12 in pregnancy that would have implications for deriving of specific reference ranges of B12 biomarkers in pregnancy, correct diagnosis and management of B12 deficiency and for defining evidence-based requirements for B12 intake in pregnancy. Thus, the study should be of great interest considering that vitamin B12 deficiency is highly prevalent among pregnant women in low- and middle-income countries with serious consequences for fetal and maternal health, and that the currently available data in this research area are very limited.  The protocol is very well written; however, in my view it would benefit if the following issues were addressed: 

**1.**  The text for the sample size estimation is very confusing. It is unclear what are the particular pharmacokinetic parameters that will be measured, what do you mean under 'clearance of B12' and what is the value of the 'geometric mean estimate of clearance'. Under the conditions of continuous vitamin B12 supplementation during the study (28 days) blood B12 concentrations would be expected to increase and maybe gradually to reach a steady state rather than going below the baseline or cleared from the circulation. Moreover, it is known that B12 is accumulated in the liver and due to its enterohepatic circulation, it is expected that B12 concentrations in plasma/serum will be maintained for some time even after the cessation of the supplementation. The reference provided for the sample size refers to drug pharmacokinetic studies in an acute setting where the maximum concentration and area under the curve are considered for the estimation but in the protocol these pharmacokinetic parameters have not been mentioned. In the ‘Statistical methods’ section is stated that ‘Steady state levels will be utilized as surrogates for clearance’ but there isn’t anything like this in the text for the sample size and how ‘steady state’ will be used as ‘clearance’ when these would go in different directions in the case of B12 supplementation. These issues need clarification.  

**2. ** Details should be provided for the laboratory methods that will be used for the assessment of vitamin B12 biomarkers: which laboratory will conduct the analysis, what are the variabilities of the methods and what will be the corresponding quality control. This information is required to support the reliability of the data that will be generated.

**3.  **In the ‘Intervention description’ section is stated that ‘Vitamin B12 doses will be provided to participants within 30 minutes following the morning meal (and evening meal for Q12H dosing)’, however justification for this approach has not been provided. It is known that the absorption of Intrinsic Factor (IF)-B12 complex in the terminal ileum is limited to 1.5-2.5 μg per meal due to saturation of the apical surface receptors and there is a refractory period of a couple of hours with restricted IF-B12 uptake. Thus, B12 content of the meal provided 30 minutes before the supplemental dose may jeopardize the absorption of B12 from the supplement and care should be given in order this meal to contain a minimum amount of the total daily B12 intake from food.

**4. ** Limited information is provided for the diet that participants will receive during the trial apart from the fact that it will be designed to contain the daily vitamin B12 requirements for pregnant women of 2.6 μg. Considering that this is a metabolic study, it would be a good practice the content of B12 in both total daily diet and supplement solution to be analytically verified in random samples throughout the different arms of the trial.

**5. ** The protocol states that ‘The main objective of the analysis is to assess the steady levels of B12 for the different dose cohorts (2.6μg, 10μg and 50 μg) in pregnant participants’. However, the duration of the intervention is 4 weeks and there is a chance that for some of the biomarkers this will be not long enough to reach a steady level. Are you going to introduce a hierarchy for the importance/reliability of the biomarkers in that scenario?

**6.  **There is a slight discrepancy in the indicated days for blood sampling on page 11 of the protocol in the subsections ‘Plans for assessment and collection of outcomes’ and ‘Plans for collection, laboratory evaluation and storage…’ that needs to be corrected.

**7. ** The Abstract states that the outcome of the trial will not be able to compare Q12H and Q24H dosing at 50 µg/day. Probably this is an error as within the limitations in the protocol the text is different as it says that this problem will be confined to ‘at doses below 50 μg’.

**8. ** There is some unclear text in the middle of the second paragraph, page 10 of the protocol that needs editing.

Is the study design appropriate for the research question?

Yes

Is the rationale for, and objectives of, the study clearly described?

Yes

Are sufficient details of the methods provided to allow replication by others?

Partly

Are the datasets clearly presented in a useable and accessible format?

Not applicable

Reviewer Expertise:

Micronutrients, B-vitamins, pregnant women, children, dietary recommendations, risk analysis

I confirm that I have read this submission and believe that I have an appropriate level of expertise to confirm that it is of an acceptable scientific standard.

# Reviewer report: Reviewer response for version 2

**DOI:** 10.21956/gatesopenres.17759.r39860

**Published:** 2025-10-24

**Authors:** Tor Strand

**Affiliations:** 1 University of Bergen, Bergen, Norway

The introduction is comprehensive and well-written, providing a clear rationale for the study. However, it lacks references to recent large randomized controlled trials (RCTs) in pregnancy that could inform the expected response to the selected vitamin B12 doses—both in terms of average effect and variability in biomarker responses. The research question is highly relevant, and the proposed design is feasible. The study has the potential to generate valuable knowledge about the pharmacokinetics of cobalamin in pregnant women in a low-income setting. Stratifying participants by baseline serum cobalamin concentration makes sense, but the justification for the chosen cut-off values could be further elaborated.

 The study includes two strata (vitamin B12 sufficient and insufficient) within which participants will be assigned to one of three daily doses: 2.6 µg, 10 µg, or 50 µg. For the 50 µg dose, participants will be randomized to receive the supplement either once or twice daily. The doses will be tested sequentially, beginning with the lowest. Altogether, the study will include 10 distinct groups based on strata and randomization, with five participants per group. However, the terminology “subgroups” in the description (“In total, there will be 10 subgroups each with 5 participants”) is confusing and should be avoided or clarified. The different stratified intervention groups are not easy to understand from the text and I turned to the trial registry to further understand the design. I therefore believe that  the 5 study arms could be described with more clarity in this protocol

 Differences in gastrointestinal function affecting the absorption of cobalamin may complicate the interpretation of pharmacokinetic. This increased variability will affect the interpretation of the results, particularly given the analytical approach (the way I understand it) particularly with regard to how the authors will interpret steady state (If the 95% confidence interval of slope would contain zero, then achievement of steady state will be made.)

 The inclusion of metabolomics, proteomics, and genomics to identify novel biomarkers of vitamin B12 status is innovative and potentially valuable. However, several methodological aspects require clarification:

- Please define the abbreviation “RCF” (or use g-force).
- Specify how biomarkers will be analyzed (e.g., immunological, microbiological, chromatographic, or combined approaches) and indicate where the analyses will be performed,.
- I suggest providing further detail on biomarker handling and analysis procedures, including sample storage and quality assurance
- The statistical analysis section is difficult to follow. This may partly reflect the reviewer’s limited background in pharmacokinetic methods; however, the text could still be made more accessible.
- The title suggests an RCT design with some degree of blinding, yet the analytical approach does not fully reflect this expectation.
- The rationale for stratification appears to be achieving balance across study groups, but it remains unclear how comparisons will be made within strata.
- The description of steady-state testing (“pre-dose B12 levels on three days during week 4 will be regressed over time…”) could be simplified or explained in more intuitive terms.
- The definition of steady state using mixed-effects regression and confidence intervals requires clearer justification.
- The sample size calculation is not fully accessible to me, and given the high biological variability of cobalamin and holoTC, the proposed sample size may be too small to meaningful describe differences (assuming that this is the purpose).
- Part of the analysis plan, particularly the comparison of fold-changes in mean steady-state B12 levels, is difficult to understand and should be rewritten for clarity.

The section on interim analysis is also confusing to me. The statement that “The serum B12 results will be reviewed with the DSMB at the completion of each dose cohort. There are no stopping rules for the study.” does not describe an interim analysis in the conventional RCT sense. Rather, this appears to refer to ongoing safety and feasibility monitoring.

 In summary, this is a scientifically important and well-motivated study addressing a key evidence gap in pregnancy nutrition research. The design is feasible and thoughtfully planned. However, clarifications on biomarker analysis, statistical methods, stratification rationale, and interim procedures could be improved.

 Is this statement in the abstract correct? “This is the first controlled feeding study to be conducted in sub-Saharan Africa.”, if not, please reconsider.

 Table 2 presents the five study groups by stratum. I suggest simplifying this figure, as I initially struggled to understand it. I also suggest that you add information about the number in each bin (n=5).

Is the study design appropriate for the research question?

Yes

Is the rationale for, and objectives of, the study clearly described?

Yes

Are sufficient details of the methods provided to allow replication by others?

Partly

Are the datasets clearly presented in a useable and accessible format?

Not applicable

Reviewer Expertise:

Epidemiology and nutritoal clinical trials

I confirm that I have read this submission and believe that I have an appropriate level of expertise to confirm that it is of an acceptable scientific standard, however I have significant reservations, as outlined above.

# Reviewer report: Reviewer response for version 1

**DOI:** 10.21956/gatesopenres.17350.r38162

**Published:** 2024-10-23

**Authors:** Kelsey Cochrane

**Affiliations:** 1 The University of Saskatchewan,, Saskatoon, Canada

Overall

- In this study, 40 pregnant women and 10 non-pregnant women will be randomized 1 of 3 doses of vitamin B12 (2.6, 10 and 50) x 4 weeks.
- Randomization will be stratified by vitamin B12 status (“sufficient/insufficient”)
- Overall, the manuscript is clear. My main comment relates to assumptions that higher status means optimal status – perhaps more measured language should be used throughout to more accurately reflect outcomes expected for this study – e.g., that it will improve our understanding of dose-response to vitamin B12 during pregnancy, and how it varies as compared to a non-pregnant state. Unless status is shown be associated with a clinical outcome, its difficult to know clinical significance – while this study may explore associations with clinical outcomes, it likely isn’t powered to prove a causative association (but can be used to inform future studies).

Abstract

- Overall the abstract is clear and well justified
- Would be helpful to add how often and when the primary endpoints (serum B12 markers) will be evaluated to the methods section. Is the primary goal to evaluate B12 status at a certain timepoint (at 4 weeks), change in status, or is it to generate a PK curve across multiple timepoints?

Background

- Well written and well justified background. Good outline of available literature.
- Might be helpful to acknowledge the limitations of “sufficient” and “insufficient” cut-offs – that these are not established/may not be meaningful in pregnancy. This further supports the need for your study.

Methods:

- I wonder if the language for objective #2 should be re-phrased – does increased status necessarily mean “improved” status? Is it possible that a decline in certain status indicators is normal in pregnancy? Something to consider re: the wording to “improve” status indicators. This also applies to the statistical methods section – can you really determine the “optimal” dose based on serum values (e.g., is highest necessarily optimal?)
- Why only single blinded? Is it possible to blind study investigators, or at minimum those doing statistical analyses/data interpretations?
- How was gestational age of 25-28 weeks determined?
- Re: exclusion criteria – what is considered “high dose” vitamin B12 supplementation? Do you expect that most participants will be routinely consuming a multiple micronutrient supplement, or is iron/folic acid supplementation only most common in this region?
- Does your sample size account for possible attrition? Have similar studies (e.g., inpatient, controlled feeding) been done in this region to get an idea of what attrition may look like? Suspect some individuals may not like the inpatient aspect, but I am not familiar with research norms in this region.
- Can a bit more detail on methods for breastmilk collection be provided – e.g., how much milk total is the study collecting – a full breast expression divided into 10 mL aliquots? What is the outcome to be evaluated in these specimens?

Is the study design appropriate for the research question?

Yes

Is the rationale for, and objectives of, the study clearly described?

Yes

Are sufficient details of the methods provided to allow replication by others?

Partly

Are the datasets clearly presented in a useable and accessible format?

Yes

Reviewer Expertise:

nutrition, B-vitamins, pregnancy, human milk

I confirm that I have read this submission and believe that I have an appropriate level of expertise to confirm that it is of an acceptable scientific standard, however I have significant reservations, as outlined above.

# Reviewer report: Reviewer response for version 1

**DOI:** 10.21956/gatesopenres.17350.r38160

**Published:** 2024-10-11

**Authors:** Yuan Jiang

**Affiliations:** 1 Department of Neuroscience, Karolinska Institutet, Solna, Stockholm, Sweden

The study protocol involves a single-blinded, stratified, dose-ranging trial in 40 pregnant women and 10 healthy controls, aiming to assess pharmacokinetics and identify the optimal dose of vitamin B12 during pregnancy in Tanzania. Participants are assigned to one of three doses (2.6, 10, and 50 µg) based on their baseline vitamin B12 status. Those receiving 50 µg are randomly assigned to receive this dose either once daily (Q24H) or twice daily (Q12H). Overall, the study is interesting and meaningful, but the authors need to provide further explanations in the following areas:

1. For the 40 pregnant participants, please clarify why the gestational age of 25–28 weeks was chosen. One of the critical functions of B12 is the prevention of fetal neural tube malformations, which form around the 6th week of pregnancy. Therefore, the significance of vitamin B12 supplementation in early pregnancy might be even more important.
2. The study includes a follow-up point at postpartum. However, it is unclear why adverse maternal and neonatal health outcomes are not included in this study. As the clinical-relevant vitamin B12 level is more important than the sufficient vitamin B12 status (the main outcome of this study), the measurement of adverse maternal and neonatal health outcomes might be more interesting.
3. The concept of “sufficient status” for vitamin B12, which is a key outcome of this study, should be clearly defined and should be provided when the term is first mentioned, rather than in the last section of the statistical methods.
4. Vitamin B12 is essential for many bodily functions. However, excessive amounts can have negative effects. Therefore, the potential side effects of achieving sufficient vitamin B12 status might be important to be recorded and analyzed.
5. The authors mention that the sample size calculation is based on achieving a pharmacokinetic parameter (i.e., clearance) of B12 within 20% precision, or 95% confidence intervals within 60% and 140% of the geometric mean estimates of clearance at 90% power. The range of 60% to 140% seems too wide to be clinically meaningful. Please provide the reference for these values and describe the power calculation in detail. The drop-off rate should also be considered.
6.  The phrase “most days” in the description “participants will receive their assigned B12 dose every day and biospecimens will be collected most days” is vague and should be avoided. Please provide a more precise description.
7. In the statistical methods section, the authors state that “to determine whether a steady state of B12 has been achieved, individual and mean longitudinal graphs of pre-dose serum B12 levels over time across the three dose cohorts will be used. The presence of a flat profile over a period of time would indicate achievement of steady state.” How will the study handle samples where a flat profile is not observed during the entire intervention period?

Is the study design appropriate for the research question?

Partly

Is the rationale for, and objectives of, the study clearly described?

Yes

Are sufficient details of the methods provided to allow replication by others?

Yes

Are the datasets clearly presented in a useable and accessible format?

Not applicable

Reviewer Expertise:

Convential epidemiology; genetic epidemiology; public health;randomized controlled trail

I confirm that I have read this submission and believe that I have an appropriate level of expertise to confirm that it is of an acceptable scientific standard, however I have significant reservations, as outlined above.
